# Supplementary material for: Comparison of pulmonary circulation parameters acquired by cardiovascular magnetic resonance with right heart catheterization and transthoracic echocardiography in patients with recent-onset dilated cardiomyopathy
Source: PLoS One. 2026 Feb 10;21(2):e0341438. doi: 10.1371/journal.pone.0341438 (PMC12890124; doi:10.1371/journal.pone.0341438)
Supplement: Attachment — Submitted filename: Response to Reviewers.docx [file pone.0341438.s004.docx]

Emily Chenette, Ph.D.

Editor-in-Chief, PLOS ONE

Cambridge, UK

Dear Editor-in-Chief,

On behalf of all co-authors, I would like to thank you for the opportunity to revise our manuscript "**Comparison of pulmonary circulation parameters acquired by cardiovascular magnetic resonance with right heart catheterization and transthoracic echocardiography in patients with recent-onset dilated cardiomyopathy**", (submission ID PONE-D-25-33529).

We would like to thank you for this opportunity and sincerely appreciate the time and expertise of both the reviewers and the editorial team.

We have carefully addressed all comments and suggestions we received. As requested, we include the revised manuscript, the point-to-point responses to the reviewer comments, and a copy of the manuscript file with the tracked changes. We believe, that the requested changes have significantly improved the clarity, scientific rigor, and overall quality of our manuscript.

We would like to update our funding information to “Supported by the AZV grant project under Ministry of Health of the Czech Republic, grant nr. NU22-02-00418 and was written at Masaryk university as part of the project “New trends and the impact of comorbidities in the diagnosis, stratification, and therapy of cardiovascular diseases" number MUNI/A/1844/2025 with the support of the Specific University Research Grant, as provided by the Ministry of Education, Youth and Sports of the Czech Republic in the year 2025.”.

We also state that the funders had no role in study design, data collection and analysis, decision to publish, or preparation of the manuscript.

We hope that the revisions meet the expectations of the reviewers and the journal.

Thank you.

Sincerely,

MUDr. Roman Panovsky, Ph.D.

St. Anne's University Hospital Brno

Pekařská 929/56, 602 00 Brno, Czech Republic

E-mail: [panovsky@fnusa.cz](mailto:panovsky@fnusa.cz)

Tel.: +420 543 18 2409

*Dear Editor,*

*We sincerely appreciate your and the reviewer's suggestions to improve our manuscript, “Comparison of pulmonary circulation parameters acquired by cardiovascular magnetic resonance with right heart catheterization and transthoracic echocardiography in patients with recent-onset dilated cardiomyopathy,” (submission ID PONE-D-25-33529). Please find below our point-by-point responses, with our answers and changes in the updated sections indicated in italics.*

**Journal Requirements:**

When submitting your revision, we need you to address these additional requirements.

1. Please ensure that your manuscript meets PLOS ONE's style requirements, including those for file naming. The PLOS ONE style templates can be found at https://journals.plos.org/plosone/s/file?id=wjVg/PLOSOne_formatting_sample_main_body.pdf and https://journals.plos.org/plosone/s/file?id=ba62/PLOSOne_formatting_sample_title_authors_affiliations.pdf

*Thank you for providing us with the templates. We have updated our revised manuscript to follow the formatting guidelines.*

2. Your ethics statement should only appear in the Methods section of your manuscript. If your ethics statement is written in any section besides the Methods, please delete it from any other section.

*Thank you for the notice. The ethics statement approval and consent to participate has been removed from acknowledgments accordingly.*

3. We note that you have indicated that there are restrictions to data sharing for this study. For studies involving human research participant data or other sensitive data, we encourage authors to share de-identified or anonymized data. However, when data cannot be publicly shared for ethical reasons, we allow authors to make their data sets available upon request. For information on unacceptable data access restrictions, please see <http://journals.plos.org/plosone/s/data-availability#loc-unacceptable-data-access-restrictions>.

Before we proceed with your manuscript, please address the following prompts:

a) If there are ethical or legal restrictions on sharing a de-identified data set, please explain them in detail (e.g., data contain potentially identifying or sensitive patient information, data are owned by a third-party organization, etc.) and who has imposed them (e.g., a Research Ethics Committee or Institutional Review Board, etc.). Please also provide contact information for a data access committee, ethics committee, or other institutional body to which data requests may be sent.

b) If there are no restrictions, please upload the minimal anonymized data set necessary to replicate your study findings to a stable, public repository and provide us with the relevant URLs, DOIs, or accession numbers. Please see <http://www.bmj.com/content/340/bmj.c181.long> for guidelines on how to de-identify and prepare clinical data for publication. For a list of recommended repositories, please see <https://journals.plos.org/plosone/s/recommended-repositories>. You also have the option of uploading the data as Supporting Information files, but we would recommend depositing data directly to a data repository if possible.

Please update your Data Availability statement in the submission form accordingly.

*Thank you for your thoughtful comment. In this matter, we contacted the Data Protection Officer (DPO) of our hospital. With his agreement, we are now able to upload the data provided that it is not traceable and, therefore, anonymized. At our institution, we work with pseudonymized data; for example, including dates of examinations would not fulfill the conditions of our DPO. Consequently, we will upload a version approved by our DPO as supplementary material. This version excludes dates, potentially identifying data, and other sensitive information that may not comply with our institution's data-handling rules.*

4. In the online submission form, you indicated that data cannot be shared publicly - eventough it is pseudoanonymized, it still contains potentially confident data about our patients and therefore we cannot share it publicly. Data are available from the International Clinical Research Center Institutional Data Access (contact via corresponding author - ass. prof. Panovský) for researchers who meet the criteria for access to confidential data.

All PLOS journals now require all data underlying the findings described in their manuscript to be freely available to other researchers, either 1. In a public repository, 2. Within the manuscript itself, or 3. Uploaded as supplementary information. This policy applies to all data except where public deposition would breach compliance with the protocol approved by your research ethics board. If your data cannot be made publicly available for ethical or legal reasons (e.g., public availability would compromise patient privacy), please explain your reasons on resubmission and your exemption request will be escalated for approval.

*Thank you for your comment. Our case now falls into number 3. In this matter, we contacted the Data Protection Officer (DPO) of our hospital and will share the anonymized data as supplementary material, as indicated in our reply to your earlier comment (number 3).*

5. We note that the grant information you provided in the ‘Funding Information’ and ‘Financial Disclosure’ sections do not match.

When you resubmit, please ensure that you provide the correct grant numbers for the awards you received for your study in the ‘Funding Information’ section.

*Thank you for noticing this mismatch. We have updated the information in both the funding information and financial disclosure subsections.*

6. Thank you for stating the following financial disclosure:

Supported by the project National Institute for Research of Metabolic and Cardiovascular Diseases (Programme EXCELES, ID Project No. LX22NPO5104) – Funded by the European Union – Next Generation EU and by AZV grant project under the Ministry of Health of the Czech Republic, grant nr. NU22-02-00418.

Please state what role the funders took in the study. If the funders had no role, please state: "The funders had no role in study design, data collection and analysis, decision to publish, or preparation of the manuscript."

If this statement is not correct you must amend it as needed.

Please include this amended Role of Funder statement in your cover letter; we will change the online submission form on your behalf.

*As suggested, we have included the statement "The funders had no role in study design, data collection and analysis, decision to publish, or preparation of the manuscript" in the cover letter.*

7. If the reviewer comments include a recommendation to cite specific previously published works, please review and evaluate these publications to determine whether they are relevant and should be cited. There is no requirement to cite these works unless the editor has indicated otherwise.

*Thank you for clarifying. We confirm that none of the reviewers suggested including any other works in our manuscript.*

**Review Comments to the Author**

**Reviewer #1:**

systolic and diastolic function and pulmonary hypertension in 84 patients with recent-onset dilated cardiomyopathy. All patients had a CMR examination, RHC (including pulmonary capillary wedge pressure (PCWP) and pulmonary vascular resistance 42 (PVR)), and TTE. The pulmonary transit time (PTT) and corrected pulmonary transit time 43 (PTTc) correlated with PCWP, cardiac index, PVR, and E/e’. ROC AUC was of 72.7% for PTT and 75.3% for PTTc, with cut-off values of 8.62 s (PTT) and 8.52 s (PTTc). The authors conclude that CMR-derived PCP might offer critical information to evaluate the cardio-pulmonary system comprehensively in patients with DCM.

The manuscript deals with an interesting topic and is based on sound methodology,

There are some points the authors might wish to address to further improve the manuscript.

*Thank you very much for your review and valuable input. We will address each of your comments below.*

1) Patients were divided in groups according to their PH and diastolic function. What was the respective rationale? Please specify.

*Thank you for the question. Our rationale for dividing patients into two groups based on pulmonary hypertension (PH) status and diastolic function was based on the distinct physiological mechanisms through which these conditions influence pulmonary transit time (PTT) and pulmonary circulation parameters (PCP).*

*For pulmonary hypertension, right-heart catheterization represents the gold standard for diagnosis, and the elevated pulmonary artery pressure values determined by this method (>20 mmHg) officially define PH. Separating patients into PH and non-PH groups allowed us to assess whether PTT and PTTc could discriminate between patients with and without elevated pulmonary pressures using an established gold-standard reference. This grouping was therefore essential to evaluate the diagnostic utility of these CMR-derived parameters.*

*For diastolic dysfunction, transthoracic echocardiography remains the most reliable noninvasive method for grading diastolic function. Diastolic function is the ability of the myocardium to relax and adjust its compliance to allow adequate and pressure-efficient ventricular filling during diastole. It is essential for maintaining optimal hemodynamic stability; therefore, it affects PCP in another way. Grouping patients by diastolic dysfunction grade allowed us to examine whether PCP and related measurements reflect these known pathophysiological differences.*

*Our approach enabled us to study the link between CMR-derived parameters and established echocardiographic markers across clinically relevant categories of dysfunction.*

2) Please provide quality assurance measures for CMR parameters (inter- and intra-observer agreement)

*Thank you for bringing this crucial point to our attention. While the automated algorithm for onset detection is deterministic (ensuring that signal intensity curve generation and onset identification are fully reproducible for any given ROI placement), variability in manual ROI placement contributes to measurement variability between observers and repeated measurements. Therefore, we randomly selected 10 cases (12% of the study population) and assessed the inter- and intra-observer reproducibility for pulmonary circulation parameters (PTB and PTT) using the intraclass correlation coefficient (ICC) (type C, two-way mixed-effects model). We added this description to the "statistical methods" section:*

*"Intra- and inter-observer reproducibility were assessed using the intraclass correlation coefficient (ICC) (type C, two-way mixed-effects model with 95% confidence intervals (CI) from ten randomly selected cases. These cases were analyzed by two readers, one of whom repeated the analysis two weeks apart. The repeatability was classified as poor (<0.5), fair (0.50 to 0.75), good (0.75 to 0.90), and excellent (0.90 to 1)."*

*Also, we report now in the "Pulmonary circulation parameters" subsection from the "results" section in the revised manuscript this information, i.e.,*

*"Pulmonary circulation parameters intra-observer reproducibility was excellent for both PTB (ICC 0.992, 95% CI 0.970-0.998) and PTT (ICC 0.988, 95% CI 0.953-0.997). Likewise, inter-observer reproducibility was also excellent for PTB (ICC 0.982, 95% CI 0.934-0.996) and PTT (ICC 0.969, 95% CI 0.883-0.992)."*

3) Is the software and algorithms used in this project freely available/transferable to other centers/machines?

*We thank the reviewer for the comment. The PTB and PTT analyses were performed using a custom Python script, which is not publicly available and not for commercial use. However, our approach is entirely transferable and can be reproduced on other systems and scanners by following the methodology described in the manuscript: motion correction of first-pass perfusion images, manual ROI placement in the RV and LV with automatic propagation across frames, extraction of signal intensity curves, onset detection, and calculation of PTB and PTT as the difference between LV and RV onset points (frame number or time, respectively). Our algorithm uses standard image processing techniques and readily available peak-detection methods in Python libraries. Centers with basic programming capabilities can implement this workflow using the detailed methodology provided.*

4) What was the time delay between the different assessments (CMR, Echo, RHC)?

*Thank you for the question. In the case of CMR and RHC, as stated in the discussion, the time between the two modalities was at most 8 days, which we consider essential, as hemodynamics can change significantly depending on factors such as the patient’s cardiac compensation, volume status, etc. As part of the statistical analysis, we also tested a subgroup with a maximum interval of only 3 days between the examinations; the results were comparable. Therefore, in the final version of the article, we kept the larger cohort with examinations performed within 8 days.*

*The mean time delay between CMR and RHC was 1.8 ± 2.1 days, and between CMR and ECHO, 4.7 ± 12.1 days. We updated the discussion section accordingly.*

5) Table 1: gender: the n is missing

*Thank you for this point and we apologize for the omission. We updated the table to include the number of available values for each parameter, and also specified the number of male patients.*

6) Table 1: “TEE” might rather the “TTE”

*Thank you for noticing this typo. The table has been updated accordingly.*

7) Table 1: the number of decimals should be equal for all numbers. One decimal might be sufficient

*Thank you for pointing this out. We updated the corresponding values in the tables to retain the proper number of significant digits.*

8) Table 1: for consistency, please also provide TTE LVEF and TR Vmax or TRmaxPG

*Thank you for your detailed point. Indeed, it improves consistency. Therefore, we updated Table 1 to include other TTE parameters, such as LVEF (%), TRPG (mmHg) (representing “TRmaxPG”), and sPAP (mmHg) as well..*

9) Since you report a correlation with IVS, please provide the respective value in table 1

*Thank you for this insightful point. In the "Pulmonary circulation parameters" subsection from the "results" section, we previously stated that “Other parameters correlating with PCP included the interventricular septum (IVS) from the tissue Doppler imaging (TDI).” This sentence meant the correlation of PCP with the TDI-derived peak systolic myocardial velocity S”, which was already shown in Table 1. To clarify this point, we updated the respective part of the results section to: “Other parameters correlating with PCP included the peak systolic myocardial velocity (S') of the interventricular septum (IVS) assessed by pulsed-wave tissue Doppler imaging (TDI).” In addition, since we listed the correlations in Table 2, we also included the correlation of this parameter in the table.*

10) What parameters was the diagnosis of RODCM based on? Signs/Symptoms, NT-proBNP, Imaging markers??

*Thank you for raising this relevant point. The definition of the RODCM throughout the literature is not standardized and lacks unification. In our article, we included patients with newly diagnosed dilated cardiomyopathy with heart failure symptoms appearing in the last six months, referred to our center based on the results from other hospitals. This is the most common definition. We updated the introduction section accordingly to include this definition.*

**Review Comments to the Author**

**Reviewer #2:**

General comments

The manuscript presents a retrospective cohort study analyzing pulmonary circulation parameters (Pulmonary Transit Beats [PTB], Pulmonary Transit Time [PTT], and corrected PTT [PTTc]) derived from cardiovascular magnetic resonance (CMR) imaging in comparison with right heart catheterization (RHC) and echocardiographic parameters, in 84 patients with recent-onset dilated cardiomyopathy. The authors demonstrate that PTT and PTTc correlate with systolic and diastolic cardiac function as well as hemodynamic indices from RHC, and that these metrics predict pulmonary hypertension (PH).

*Thank you very much for your review and valuable input. In addition to your comments, we also addressed those from the editor and another reviewer to our updated manuscript.*

Specific comments

1. Table 1 Formatting

- Please avoid reporting unnecessarily precise values, e.g., heart rate of 77.67 ± 17.08. Rounding should be applied where appropriate.

*Thank you for your suggestion. We updated the tables to include only the proper number of significant digits..*

- Add a column indicating the number of available data points for each parameter. If all values are complete, mention this briefly in the Methods section.

*Thank you for mentioning this point. We added the information to the table where possible. In some cases, the data points werenot available for all patients. For example, E/A data were not available for all patients because patients with atrial fibrillation lack an “A” wave.*

- Present mean and systolic pulmonary artery pressure as well as cardiac index (CI) in Table 1. The data for mPAP and CI are already presented on page 11, line 183.

*Thank you for this valuable suggestion. We updated Table 1 and added information on systolic pulmonary artery pressure (sPAP) and CI.*

- Define “pulmonary artery pressure index” in the Methods section.

*Thank you very much for noticing this omission. In Table 1, “PAPi” is defined as the “pulmonary artery pressure index,” but instead it should have been “Pulmonary Artery Pulsatility Index.” We are a transplantation + LVAD center, and PAPi is a clinically validated hemodynamic parameter used to assess the RV function, which is crucial in patients before LVAD implantation. It is defined as: PAPI = (Pulmonary artery systolic pressure – Pulmonary artery diastolic pressure) / Right atrial pressure. The methods section and table have been updated accordingly. We apologize for this oversight.*

- DPG refers to “diastolic pressure gradient,” not “Diastolic Pulmonary Artery Pressure” (page 11, line 174). DPG is the difference between pulmonary artery diastolic pressure and pulmonary capillary wedge pressure. Please provide this definition in the Methods.

*Thank you for noticing this oversight. We have added the definition in the methods section.*

2. Subgroup Division and Classification (Page 11, lines 184–187)

The numbers cited for PH subgroups do not add up to the total of 84 patients. Please verify and correct the classification.

- Remove the 28 “precapillary” entry; do not sum isolated precapillary patients with combined post- and precapillary PH.

- One patient remains unclassified; please clarify.

The correct breakdown should be:

- 37 patients without PH

- 47 patients with PH

--6 with precapillary PH.

--40 with postcapillary PH

---18 isolated postcapillary PH (IpcPH)

---22 combined post- and precapillary PH (CpcPH)

*Thank you for raising this valuable comment. The PH and non-PH subgroups consisted of 47 and 37 patients, respectively, for a total of 84 patients. We believe the issue arises from a lack of clarity in the description we provided previously. Therefore, we revised the sentence for greater clarity. Including the “precapillary” category was indeed confusing; the original rationale was primarily statistical, but from a clinical perspective, it added ambiguity, so we removed it. The same applied to patients with postcapillary PH; therefore, we excluded this category as well. We retained only “isolated precapillary PH,” “isolated postcapillary PH,” and “combined pre- and postcapillary PH”, which also better reflects the guideline-based classification. Regarding the numbers, one patient had an mPAP of 21 mmHg, a PCWP of 13 mmHg, and a PVR of 1.8 WU, and thus could not be assigned to any category. In the literature, such cases are described as either “unclassified pulmonary hypertension” or “borderline/mildly elevated mPAP without a confirmed pre-capillary or post-capillary component.” The updated description is:

 “The patients were divided into subgroups according to the signs of PH: 47 had signs of PH (PH group), and 37 had no signs (non-PH group). Among the 47 patients with PH, 6 had isolated precapillary PH, 18 had IpcPH, and 22 had CpcPH. In one particular case, the patient had elevated mPAP over 20 mmHg, therefore signs of PH, but neither elevated PCWP nor PVR; thus, the patient could not be assigned to any category.”*

3. Please provide a more detailed characterization of the patients classified as having precapillary pulmonary hypertension. Specifically, did these individuals have underlying lung disease or other identifiable etiologies for precapillary PH? Alternatively, did they exhibit features typical of postcapillary PH, apart from the absence of PAWP elevation? Additionally, please report the average pulmonary artery wedge pressure (PAWP) for this subgroup.

*Thank you for the opportunity to explain these details better. There were 6 patients classified as having precapillary PH in total. They showed a little bit higher LVEF than the whole population (27.1%* ± 9.4 vs. 24.4% ± 9.3), comparable mPAP (23 mmHg ± 1.2 mmHg vs. 22.4 ± 8.6 mmHg) and lower PCWP (12.83 ± 0.9 mmHg vs. 14.3 ± 8.6 mmHg)*. The average pulmonary artery wedge pressure for subgroups with isolated postcapillary PH was even higher - 20.3* ± 5.1 mmHg*. Additionally, from the 6 patients with precapillary PH, 3 were diagnosed with lung diseases at the time of the RHC; one patient was a smoker and drug user with high probability of underlying lung disease and in 2 cases the cause was unknown; therefore, idiopathic. Apart from the heart failure itself, they did not exhibit features typical of postcapillary PH.*

*We updated the results section to clarify this:
Of the six patients with precapillary PH, three were diagnosed with underlying lung disease at the time of the RHC; one patient was a smoker with a high likelihood of undetected lung pathology, and in two cases the cause remained unknown.”*

4. Can you provide a table divided by patients with no PH, pcPH and prePH illustrating all CMR and RHC parameters?

*Thank you for this suggestion. Yes, we can provide this table as a supplementary file. It illustrates the main CMR and RHC parameters for our cohort, and we hope it strengthens our findings.*

5. Completeness of Table 1 (Page 11, line 188)

Table 1 does not present a complete list of evaluated parameters. Please ensure all relevant variables are included or adjust the related text accordingly.

*Thank you for this suggestion. We updated the table with more evaluated parameters - including parameters such as LVEF (%), TRPG (mmHg) (representing “TRmaxPG”), sPAP (mmHg), mPAP (mmHg), and CI (L/min/*m^2).

6. Discussion

- The first two discussion paragraphs are repetitive. Please streamline and avoid using nearly identical sentences.

- The discussion is brief. Please elaborate on the clinical advantage of assessing PTB, PTT and PTTc by CMR compared to standard echocardiographic evaluation of PH.

State whether PTT or PTTc can predict increased pulmonary artery wedge pressure (PAWP), as this would markedly increase the clinical utility of your results.

*Thank you for this comment. We have made an effort to improve the discussion section by merging the first two paragraphs, expanding it, and avoiding repetition. In addition, we further expanded it by adding the clinical relevance of these parameters. Similarly to what we described previously (1), although still limited, current data show a strong predictive value for the prolongation of PCP across various cardiac diagnoses, making this dynamically developing topic attractive for clinical applications today. From a pathophysiological point of view, longer times correspond to impairments of either the pumping function of the heart (systolic for both ventricles, diastolic for the left ventricle) or the lungs’ response (foremost PH). This behaviour implies that these parameters have a unique potential to assess the cardiopulmonary system as a whole. To date, no CMR-based method reliably estimates markers of pulmonary hypertension, and even the assessment of diastolic function remains highly limited. This approach, combined with the simplicity of their acquisition and the growing body of evidence supporting their predictive value, makes them a highly promising marker for the future of integrated cardiopulmonary diagnostics.*

*Regarding your last question, PCP (particularly PTT and PTTc) correlates with PCWP and also with PVR, although to a lesser extent. In our dataset, the cohort consisted of patients with newly diagnosed HF referred to our center, and as expected, the largest subgroup was those with CpcPH. Partly because of this, we were unfortunately unable to distinguish isolated postcapillary PH using PCP alone reliably.*

*(1) Opatřil L, Panovský R, Mojica-Pisciotti M, et al. Stress and Rest Pulmonary Transit Times Assessed by Cardiovascular Magnetic Resonance. Cardiology in Review.:10.1097/CRD.0000000000000495. doi:10.1097/CRD.0000000000000495

We updated the “discussion” section accordingly.:
“Our findings suggest that the prolongation of PCP strongly correlates with parameters measured by CMR, RHC, and TTE, such as. PVR, E/e´, LVEF, PCWP, CI, and grade III diastolic dysfunction, demonstrating the ability of PTT to differentiate patients with PH using CMR. Similarly to what we have described previously (1), although still limited, current data show a strong predictive value for the prolongation of PCP across various cardiac diagnoses, making this dynamically developing topic attractive for clinical applications today. From a pathophysiological point of view, longer times correspond to impairments of either the pumping function of the heart (systolic for both ventricles, diastolic for the LV) or the lungs' response (foremost PH). This behaviour implies that these parameters have a unique potential to assess the cardiopulmonary system. To date, no CMR-based method reliably estimates markers of PH, and even the assessment of diastolic function remains highly limited. This approach, combined with the simplicity of their acquisition and the growing body of evidence supporting their predictive value, makes them a highly promising marker for the future of integrated cardiopulmonary diagnostics.”

We also updated the “study limitation” paragraph:
“ This study has some limitations. Foremost, it is a retrospective single-centre study. Another limitation is our patient selection, RODCM. In them, isolated precapillary PH is rare; therefore, data on comparing precapillary with other types of PH are severely limited. One of the objectives of this study was to determine whether PCP can distinguish patients with isolated pre- or postcapillary PH. Although these parameters (particularly PTT and PTTc) correlate with PCWP, they also show a weaker correlation with PVR. In our dataset, the cohort consisted of patients with newly diagnosed HF referred to our center, and as expected, the largest subgroup was those with CpcPH. In contrast, the number of patients with isolated precapillary PH was very limited. Partly because of this, we were unfortunately unable to differentiate isolated pre- or postcapillary PH using PCP alone reliably. It would also be invaluable to study prognosis in RODCM patients based on PCP, but we were not able to do so based on our data. However, we plan to carry out such a study in the future.”*
